# Stem cell dysfunction and rejuvenation strategies in ageing: emerging advances in regenerative medicine

**DOI:** 10.3389/fcell.2026.1830358

**Published:** 2026-05-26

**Authors:** Khuzin Dinislam, Anas Shamsi, Syed Tasqeruddin, Saleha Anwar, Moyad Shahwan

**Affiliations:** 1 Department of General Chemistry, Bashkir State Medical University, Ufa, Republic Of Bashkortostan, Russia; 2 Centre of Medical and Bio-Allied Health Sciences Research, Ajman University, Ajman, United Arab Emirates; 3 Department of Pharmaceutical Chemistry, College of Pharmacy, King Khalid University, Abha, Saudi Arabia; 4 Center of Excellence in Precision Medicine and Digital Health, Department of Physiology, Faculty of Dentistry, Chulalongkorn University, Bangkok, Thailand

**Keywords:** ageing, clinical trials, regenerative medicine, rejuvenation approaches, stem cell therapy, stem cells

## Abstract

Ageing is a complicated phenomenon that is defined by the progressive decline in the body’s functions that leads to weakened regenerative potential and greater vulnerability to various age-related diseases. There is evidence that highlights that stem cell dysfunction and exhaustion are the primary reasons for tissue degeneration that progresses with age and hence provide aid to regenerative therapeutic approaches. Stem cells have shown the ability to influence age-related cellular dysfunction in preclinical models mainly via paracrine signaling, immune modulation and tissue repair processes. Nonetheless, clinical evidence primarily confines to early-phase trials, showing inconsistent results based on cell type, delivery method and disease context. Several types of stem cells are being studied rigorously for their regenerative, immunomodulatory, and anti-ageing properties. Pre-clinical and early-phase clinical studies suggest potential benefits of stem cell-based interventions in musculoskeletal, cardiovascular and neurodegenerative disorders although effect sizes vary and long-term efficacy remains under investigation. Furthermore, emerging technologies such as tissue reprogramming, senolytics and niche modulation also contribute to improving therapeutic strategies. This review aims to provide a comprehensive overview of the existing knowledge of stem cell biology and therapies from various preclinical and clinical studies, along with rejuvenation approaches.

## 1 Introduction

Ageing and regeneration are the most fundamental biological processes of the body. Ageing, being the complex one, is a multifaceted phenomenon, characterized by the progressive decline in the body’s physiological and cellular functions, resulting in greater susceptibility to age-related diseases (Chu et al., 2024). The global population above 60 years is expected to be doubled from 12% (900 million) to 22% (2 billion) between the years 2015 and 2050, with an estimated life expectancy of 73 years. This increase in human life span led to demographic re-distribution with a disproportionate increase in those people who are over 70 years old and ultimately resulted in countries that have more than 1/5th of their population with age greater than 70 years (Garmany et al., 2021; Officer et al., 2020). As we already know, ageing contributes to various diseases, including all non-communicable diseases, neurodegenerative diseases, and musculoskeletal disorders (Li et al., 2021). Thus, ageing poses major health challenges and hence strains the systems, as 71% of mortality is from non-communicable diseases, with 58% mortality data from people over 70 years of age (Garmany et al., 2021). A longer, healthier life expectancy often leads to an increase in chronic diseases, mostly affecting the older population. Multimorbidity worsens the condition and makes the treatment and care management more difficult, leading to expensive healthcare facilities. Around 1.3 billion people from the globe live with major disabilities, while the middle- and low-income countries are facing similar demographic changes that unknowingly put more pressure on the underdeveloped healthcare infrastructure (Gianfredi et al., 2025). Thus, it can be concluded that ageing is no longer just a personal but a major global health crisis.

Physiological and pathological alterations are a part of ageing. Physiological alterations are defined as the degradation processes that occur post-maturation. DNA damage, shortening of telomere, mitochondrial dysfunction, impaired autophagy, and loss of nicotinamide adenine dinucleotide (NAD^+^) are some of the examples of physiological changes, leading to weakened systemic functions. In contrast, pathological changes are those changes that happen due to various extrinsic factors, including non-communicable diseases, neurodegenerative diseases, degenerative joint diseases, and multi-organ failure. These impairments and alterations lead to DNA damage and genomic instability, which include the two major characteristics of the ageing process. This highlights the urgency for strategic treatment approaches that address the process of ageing rather than simply treating its symptoms (Li et al., 2024; Zhang L. et al., 2023; Zhang et al., 2025). Additionally, the resident stem cells and progenitor cells present in the ageing tissues get depleted and become non-functional, which affects the natural process of regeneration. Simultaneously, the senescence cells create a chronic inflammatory environment by gathering and releasing proinflammatory cytokines, which are known as senescence-associated secretory phenotypes (SASP), which further accelerate tissue deterioration (Guo et al., 2022; Picerno et al., 2021). A recent study also suggested efforts to delay ageing process, reduce functional tissues and increase healthy life span by using novel therapeutic approaches (Yu et al., 2025). One such strategy is the emerging field of regenerative medicine and stem cell-based therapies, which represents a transformational horizon to manage the age-related decline and enhance life expectancy.

The researchers are aiming to restore physiological integrity and function by harnessing those cells that can replace, rejuvenate, or regenerate damaged cells or tissues, thereby addressing the root cause of age-related decline (Velikic et al., 2024). Regenerative therapeutic approaches include cellular therapies, bioengineered tissues, and anti-senescence strategies (Garaci et al., 2025; Meretsky et al., 2024; Saliev and Singh, 2024). Specifically, stem-cell-based therapies are leading the way. Stem cells are capable of self-renewal and differentiation into specialized cell types and hence making them ideal for the rejuvenation of lost or aged cells. Stem cells have the potential to reverse organ dysfunction rather than simply treating late-stage disease by restoring normal cellular composition and function (Hussen et al., 2024). According to recent advances, anti-senescence and regenerative technology are major key tools for bridging the nine-to 10-year gap between longevity and disease-free health span (Garmany et al., 2021). For instance, a combination of senolytic drugs and cell therapies is being investigated to revive aged organs and such approaches reframe the healthcare “from care to cure” of ageing-associated diseases (Blau and Daley, 2019; Braunwald, 2018; Cossu et al., 2020; Kirkland and Tchkonia, 2020). In regenerative medicine, stem cells can be used for two major reasons: (a) cell-based therapy to replace or repair damaged cells or tissues (b) disease modeling or drug discovery (Pirsadeghi et al., 2024; Sadiq et al., 2025). This review aims to comprehensively investigate the therapeutic potential of stem cell-based therapies for age-related conditions, with emphasis on elucidating the underlying mechanisms, evaluating current applications, addressing existing challenges and limitations as well as future perspectives.

## 2 Hallmarks of ageing

Ageing is characterized by a series of biological processes contributing to diminished physiological function and enhanced susceptibility to diseases as organisms age (Guo et al., 2022). The framework of hallmarks of ageing offers a conceptual basis for comprehending biological decline across various tissues. Initially defined by López-Otín et al., 2013 and subsequently broadened to encompass more interrelated processes, these hallmarks collectively impact stem cell activity, tissue repair and organismal ageing (López-Otín et al., 2013; López-Otín et al., 2023). Rather than functioning independently, these mechanisms act as a cohesive network that gradually reduces regenerative ability, especially in stem cell compartments (Esposto and Balendra, 2022; Hambright et al., 2020; Schmauck-Medina et al., 2022).

### 2.1 Genomic instability

Genomic instability and accumulation of DNA damage a primary hallmark of ageing, which contributes to cellular dysfunction and diseases (González-Meljem et al., 2018). Genomes are unstable due to the continual induction of DNA damage, primarily from endogenous sources. It is noteworthy that DNA damage differs from DNA mutations. DNA damage causes physical changes in the structure of nucleic acids, such as breaks, crosslinks, depurination, depyrimidination, and base changes, whereas mutations occur as errors during the DNA repair or replication process, or sometimes spontaneously (Vijg and Montagna, 2017). Therefore, genomic instability is referred to as a range of DNA modifications, including point mutations, chromosomal rearrangements, deletion and insertion mutations and whole chromosome numerical alterations which irreversibly change the genome information content, contributing to genomic instability (Aghamir et al., 2025). Genomic instability contributes to the compromise of cell function and can trigger cancer, as well as cell loss (Maldonado et al., 2023). Stem cells rely on intact genomes for renewal, so genomic instability severely limits their regenerative capacity (Tsai, 2016).

### 2.2 Telomere attrition

Telomeres are the genomic segments located at the ends of the linear chromosomes. The shortening of these telomeres, serving as a cap on each arm of all chromosomes, contributes to cellular senescence and ageing (Eppard et al., 2023). According to the telomere hypothesis, senescence occurs when telomeres in somatic cells reach a critical length known as the Hayflick limit (Hayflick, 1965; Hayflick and Moorhead, 1961; Hirt et al., 2013; Razgonova et al., 2020). The loss of nucleotides in the telomeric region disrupts the T-loop and opens the exposed overhang to the DNA damage response (DDR), as the telomere is now recognized as a double-strand break (DSB), eventually leading to senescence and apoptosis (Harley et al., 1990; Shay, 2018; Verdun et al., 2005). Research also indicates that the shortening of telomere leads to DDR activation and cellular senescence in various animal models resembling aspects of ageing and age-associated diseases (Rossiello et al., 2022). Despite a halt in the telomere attrition process due to cell cycle arrest, the chronic inflammatory environment created by SASP can contribute to the slow progression of various age-related conditions (Coppé et al., 2008; Cuollo et al., 2020; Libby et al., 2009; Shakeri et al., 2018). This trend may occur due to the negative impacts on other cells in the microenvironment, for instance, increased reactive oxygen species (ROS) generation and senescent cell accumulation (Fossel et al., 2022; Sahin and DePinho, 2012).

### 2.3 Epigenetic alterations

One of the most preserved signs of ageing is epigenetic alterations that include DNA methylation, histone alterations, chromatin remodeling and noncoding RNAs. Various biological processes and markers play crucial roles in the developmental process of ageing, but epigenetic changes stand out due to their vital role in gene regulation and cellular identity (Ermolaeva et al., 2018; Izadi et al., 2024). The process of methylation occurs because of the transfer of a methyl group from S-adenosyl methionine (SAM) to the fifth carbon present in cytosine to form 5-methylcytosine by the catalytic action of three DNA methyltransferases (DNMTs) called DNMT1, DNMT3a, and DNMT3b (Xiao et al., 2019). Post-translational histone changes can control the ageing process and either stimulate or suppress gene expression. ADP ribosylation, methylation, acetylation, ubiquitination, and phosphorylation are some examples of histone modifications. The most extensive research on those known to impact ageing is methylation and acetylation at lysine residues.

On the other hand, chromatin is a flexible and dynamic entity consisting of DNA and histones, which can exist as either euchromatin or heterochromatin (Nothof et al., 2022). The term ‘chromatin remodeling’ refers to a set of nuclear architecture alterations that occur throughout the genome and are discernible at the level of individual chromosomes or chromosomal domains like centromeres. Cellular senescence has been found to cause significant chromatic structural remodeling that ranges from modifications and changes in histone components to changes in chromatin compartments and topologically associating domains (TADs) (Chandra et al., 2015; Liu et al., 2022; Zhao and Chen, 2022). From yeast to human, the global canonical histone loss is thought to be a typical aspect of ageing (Dang et al., 2009; Hu et al., 2014; O’Sullivan et al., 2010). Finally, non-coding ribonucleotides have a significant role in regulating epigenetic modifications. MicroRNAs are endogenous non-coding RNAs that can inhibit gene expression via post-translational alterations. Various evidence suggests that miRNAs regulate other ageing processes, such as mTOR, AMPK, and sirtuin pathways (Jin Jung and Suh, 2012; Turko et al., 2025). In 2018, a systematic study found that miR-125b regulates MAPK, which is linked to Alzheimer’s dementia and early life stress (Lemche, 2018). Thus, changes in miRNA expression can be a sign of early ageing and can help us recognize various age-related disorders like atherosclerosis, Alzheimer’s disease (AD) and cancers.

### 2.4 Loss of proteostasis

Mammalian cells regulate the process of protein synthesis, protein folding, their transport, post-translational changes and their destruction to maintain protein equilibrium. This entire process comes under proteostasis or protein homeostasis. Any impairment or loss of protein homeostasis can lead to ageing, and therefore, it is considered a hallmark of ageing. By eliminating aberrant proteins through ubiquitin molecules, the multi-subunit proteolytic complex known as proteasome plays a critical role in preserving mammalian cellular proteostasis (Tang and Xiao, 2023). Numerous studies show a link between disrupted protein homeostasis and ageing that ultimately contributes to the progression of age-related diseases (Cai et al., 2022). Protein quality control (PQC) also contributes to maintaining a healthy proteome, necessary for cellular homeostasis. However, along with ageing progression, the efficiency of PQC pathways to regulate proteostasis weakens, and hence, the rise of hazardous misfolded proteins increases within the cell (Kumar et al., 2023). The disruption of PQC pathways is a significant sign in the pathogenesis of neurodegenerative diseases like AD, PD, amyotrophic lateral sclerosis (ALS), and Huntington’s disease (HD) (Ruz et al., 2020).

### 2.5 Disabled macro-autophagy

Autophagy, a Greek word meaning self-eating, is a catabolic process that is very crucial for the normal functioning of the body as it recycles many cellular processes like excess constituents, abnormally accumulated or damaged macromolecules, organelles and pathogens, that are no longer required by the body or may cause harm to the body. It is a protective pathway to maintain cellular equilibrium, and its maintenance is necessary for longevity. Dysregulation of autophagy is a primary factor in the development of various age-related diseases (Nieto-Torres and Hansen, 2021). Many studies across different organisms have shown that reduced autophagy contributes to ageing, with cells from long-lived animals and humans having elevated autophagy levels (Hansen et al., 2018; Leidal et al., 2018). Studies in *Caenorhabditis elegans*, *Drosophila*, rats, and human cells have shown that lysosomal proteolytic function declines with age, impairing autophagic flux and hence contributing to the development of age-related diseases. In addition, along with molecular chaperones and ubiquitin proteasome system (UPS), autophagy plays a role as a central regulator of cellular proteostasis. In line with this, genetic manipulation of core autophagy machinery components or regulators increases age-related protein aggregation, shortens lifespan, and exacerbates clinical characteristics in animal models (Aman et al., 2021).

### 2.6 Dysregulated nutrient sensing

Nutrient sensing is a biological process by which an organism detects changes in nutrient availability via dedicated biochemical sensors or surrogate factors and responds adaptively to preserve energy and nutrient homeostasis (Graupera and Claret, 2018). Nutrient-sensing pathways (NSP) play a vital role in the ageing process. For example, restricting dietary intake can increase the longevity of many organisms by suppressing nutrient sensing. In contrast, dysregulation of nutrient sensing is frequently reported in elderly individuals and age-related diseases such as type 2 diabetes (T2D) (Yu et al., 2024). These NSPs work as a link between ageing and diet. Modifications in these pathways have emerged as a new research topic in ageing, as they can be modulated both pharmacologically and by dietary treatments. Some NSPs that are impaired by ageing are IGF1/PI3K/AKT/mTOR and AMPK/Sirtuin/PGC1α. These pathways regulate protein synthesis, cell cycle, autophagy, DNA replication, stress response and glucose homeostasis. Recent studies also suggest that microRNAs play a crucial role in influencing nutrient-sensing pathways (Micó et al., 2017).

### 2.7 Mitochondrial dysfunction

Mitochondria are crucial for health and diseases, regulating energy production through ATP generation, hormone production and neurotransmitter production. It also plays a vital role in shaping communication by interacting with organelles and the environment (Casanova et al., 2023). Any alteration in mitochondrial function can significantly impact ageing and chronic age-related diseases as mitochondria regulate cellular energy, oxidative balance, and calcium levels. During dysfunction, mitochondria exhibit decreased ATP production, changes in apoptosis regulation, increased ROS production and impaired calcium signaling (Nehlin, 2023; Norat et al., 2020). The accumulation of mutations in mitochondrial DNA (mtDNA) is the principal cause of mitochondrial anomalies, which contribute to ageing and associated disorders (Kujoth et al., 2007; Paraskevaidi et al., 2017). Increased ROS generation and mitochondrial apoptotic pathway activation in aged cells contribute to systemic degradation of numerous tissues, resulting in overall organ and tissue functions, like the heart, liver, brain, and muscle. Moreover, dysfunction in mitochondrial proteostasis is also associated with altered cellular activities in aged cells (Somasundaram et al., 2024).

### 2.8 Cellular senescence

Cellular senescence is a steady growth arrest caused by events like DNA damage, telomere dysfunction, organelle stress and oncogene activation. Hayflick and Moorhead first reported senescence in human fibroblasts in 1961. Senescence prevents uncontrolled cell growth but also contributes to the process of ageing by reducing tissue regeneration (Alcorta et al., 1996; Hayflick, 1965; Hayflick and Moorhead, 1961; Serrano et al., 1993). Furthermore, accumulation of senescent cells secretes inflammatory factors and hence disrupts tissue functions as well (González-Meljem et al., 2018). Characteristics of senescence include increased β-galactosidase activity, enhanced cell cycle inhibitors, chromatin alterations and SASP, which causes inflammation and tissue failure. While senescence inhibits tumors and helps in wound healing, its persistence contributes to chronic age-related diseases. Experiments on transgenic mouse models also indicate that eliminating senescent cells delays ageing and age-related disease progression (Di Micco et al., 2021).

### 2.9 Stem cell exhaustion

Stem cell exhaustion refers to the age-related decline in the number, self-renewal and regenerative potential of tissue stem and progenitor cells that reduce tissue maintenance and repair, thereby contributing to the age-related pathologies (Ermolaeva et al., 2018; Hambright et al., 2020). This depletion emerges from DNA damage, telomere attrition, epigenetic modification, metabolic and proteostasis dysfunction, chronic inflammation and altered systemic signals that regulate the aberrant activation and loss of quiescence. Studies demonstrated that both cell intrinsic damage and extrinsic changes together reduce the stem cell functions, while interventions targeting metabolism and other causative factors can partially restore the regenerative abilities of these stem cells (López-Otín et al., 2013; López-Otín et al., 2023; Mi et al., 2022; Picerno et al., 2021).

### 2.10 Altered intercellular communication

Altered intercellular communication is a significant characteristic marker of ageing, which influences numerous biological processes and contributes to the functional decline associated with ageing. This impairment in intercellular communication can occur due to alterations in various mechanisms that include alterations in SASP, modifications in ligand-receptor interactions, and changes in extracellular vesicles (EVs) composition, either by elevating or mitigating the phenomenon of ageing (Tenchov et al., 2023). Along with pro-inflammatory cytokines, SASP constitutes proteases and growth factors as well, and altogether they contribute to tissue dysfunction and poor intercellular communication, thereby promoting age-related conditions (Ribeiro-Rodrigues et al., 2023). EVs are composed of microvesicles and exosomes, which are crucial mediators of intercellular communication as they contain proteins, RNAs and microRNAs. Age-related modification in EVs composition includes abnormal protein aggregation, and modified microRNAs can alter cellular homeostasis and proteostasis (Bhunia and Kasturi, 2024; V. Wagner and Keller, 2024). EVs can either propagate damage or facilitate the repair mechanism process, depending on their cargo and their release (Romero-García et al., 2023). Additionally, modifications in ligand receptor interaction, which are very common in ageing, can also affect the immune response and tissue regeneration process (Chen et al., 2024; García-Domínguez, 2025).

### 2.11 Inflammageing

Inflammageing, also referred to as chronic inflammation, is a significant cause of ageing, characterized by a persistent low-grade inflammatory condition that contributes to various age-related diseases. Chronic inflammation is widespread across almost all major diseases, as it is associated with immune dysfunction, dysbiosis, and cellular senescence. This created a cyclical association that alleviated the decline in health among the elderly population (Andonian et al., 2024; Baechle et al., 2023). Decreased thymic production and increased immune cell senescence are caused due to ageing, which ultimately leads to chronic inflammation (Saavedra et al., 2023). One study indicated that the other hallmark of ageing, dysbiosis, allows inflammatory factors to enter tissues and thus contributes to worsening of the inflammatory process (Tan and Norhaizan, 2021). Similarly, the association of altered nutrient sensing and chronic inflammation further increases the chances of age-related dysfunctions. Inflammageing also contributes to greater risks of cardiovascular diseases, neurodegenerative diseases, and cancer. Systematic inflammation also plays a crucial role in increasing mortality risk, as it correlates with biological ageing markers such as DNA methylation and age acceleration. Despite the notion that chronic inflammation is the key cause of ageing, some evidence suggests this is not always true and that it may differ across groups (McDade, 2023).

### 2.12 Dysbiosis

An imbalance in the composition of the gut microbiome is known as dysbiosis. It is linked to both ageing and systemic inflammation (Schmauck-Medina et al., 2022). Based on some studies, it is suggested that ageing might lead to a decline in good bacteria and microbial diversity, which can worsen age-related problems by increasing pathobionts (Kelso, 2024). According to D’Amico et al. (2024), centenarians exhibit a more resilient and diversified microbiome, which is considered one of the specific microbial fingerprints related to ageing. Dysbiosis can also cause inflammation, which can further influence conditions such as gastrointestinal disorders (Mathur et al., 2024; Ragonnaud and Biragyn, 2021). Therefore, altering the gut microbiome by dietary and lifestyle modifications can provide several treatment approaches to reduce dysbiosis and related health risks in older adults (D’Amico et al., 2024; Ragonnaud and Biragyn, 2021).

## 3 Stem cells

The human body contains unspecialized cells called stem cells. They have the capacity to self-renew and can differentiate into any type of cell. Both adult cells and embryos contain stem cells. The process of specialization involves multiple steps. A unipotent stem cell cannot differentiate into as many different types of cells as a pluripotent one, since its developmental power decreases with each stage. Stem cells are broadly classified based on differentiation potential and tissue origin (Poliwoda et al., 2022; Shah and Khan, 2021; Shah et al., 2023). The classification helps in understanding their biological roles and therapeutic applications. Based on potency, there are (i) totipotent stem cells. These cells are present at the early stages of embryonic development (Gabr and El-Kheir, 2023); (ii) pluripotent stem cells, that include embryonic stem cells (ESCs) and induced pluripotent stem cells (iPSCs) and are capable of differentiating into nearly all cell types (Ahmed Al-Anazi, 2020; Mesfin et al., 2023; Romito and Cobellis, 2015); (iii) multipotent stem cells like mesenchymal stem cells (MSCs), can differentiate into a limited range of cell types (Ahmed Al-Anazi, 2020; Sobhani et al., 2017); (iv) oligopotent stem cells can differentiate into only a few cell kinds and (v) Unipotent stem cells, that call replicate and replace senescent cells but are capable of differentiating into a single cell type (Ahmed Al-Anazi, 2020; Mesfin et al., 2023). The classification based on origin or source divides the stem cells into five primary types: mesenchymal stem cells (MSCs), hematopoietic stem cells (HSCs), neural stem cells (NSCs), induced pluripotent stem cells (iPSCs) and embryonic stem cells (ESCs). While the classical classification remains foundational, the functional relevance of these categories lies majorly in their regenerative capacity, immunomodulatory properties and niche interactions, features that directly influence ageing related tissue decline (Clevers, 2015; Weissman, 2015).

### 3.1 Mesenchymal stem cells (MSCs)

Mesenchymal Stem Cells (MSCs) were first characterized as adherent stromal progenitors which are multipotent, non-hematopoietic stem cells and have the potential to differentiate into several mesodermal forms, including adipocytes, osteoblasts, and chondrocytes (Friedenstein et al., 1970). They can also differentiate into endodermal and ectodermal lineages, demonstrating their vast differentiation potential in stem cell therapeutics, thereby making it a highly recommended and promising approach in regenerative medicine. It also possesses the ability to immunomodulate and self-renew (Lykov, 2023; Slaba, 2024). The therapeutic efficiency of MSC is mediated by the release of bioactive molecules such as cytokines, growth factors, and extracellular vesicles (EVs), which play critical roles in modulating the local cellular environment, promoting cell survival, tissue repair and angiogenesis, as well as exerting anti-inflammatory properties. MSCs can even interact with immune cells, including T-cells, B-cells, macrophages, and dendritic cells, to modulate the immune responses through direct cell-to-cell interactions and immunoregulatory chemicals and can inhibit inflammation (Han et al., 2025; Vohra and Arora, 2023). MSCs have also shown promise in treating conditions like liver fibrosis, diabetes, spinal cord injuries and COVID-19 (Aulia et al., 2024). And currently, they are being actively investigated for their potential as regenerative medicines to bring change to the medical world (Slaba, 2024).

### 3.2 Hematopoietic stem cells (HSCs)

Hematopoietic stem cells (HSCs) are multipotent adult stem cells capable of long-term self-renewal and differentiation into numerous mature blood cells, including myeloid lineage and lymphoid lineage cells (Till and McCulloch, 1961; Weissman, 2000; Lee and Hong, 2019; Tang et al., 2023). Since their functions are reduced with age, these HSCs can cause various health issues and hence are very critical to the ageing phenomenon. According to studies, HSCs increase their regenerative capacity, thereby reducing efficiency and, ultimately, declining in function as age increases. All these processes lead to the progression of various hematological diseases and lowered immunological responses (Fujino et al., 2022; Li X. et al., 2025). Also, ageing results in impaired self-renewal and reduced differentiation potential of HSCs, ultimately leading to a shift towards the myeloid lineage at the expense of lymphoid cells. Some factors, like oxidative stress and epigenetic modifications, can also contribute to the HSCs’ ageing process (Chatterjee and Thakur, 2021; Shevyrev et al., 2023). Niche ageing can also influence the cells, for instance, the bone marrow microenvironment, which can also age, thereby affecting the functions of HSCs and resulting in chronic inflammation, which exacerbates age-related diseases (Shevyrev et al., 2023; Zhang et al., 2024). Recent studies also showed that transplanting young HSCs into older mice reduces ageing traits, indicating a possible therapeutic pathway. Furthermore, platelet factor 4 (PF4) also plays a crucial role in sustaining HSCs’ function and declines with age. Restoring PF4 levels in aged mice has shown promising benefits for regenerating HSCs (Wang et al., 2025; Zhang et al., 2024).

### 3.3 Neural stem cells (NSCs)

Neural stem cells (NSCs) are stem cells found in the nervous system and, during development, form the nervous system. In adults, very few NSCs persist and are generally dormant; yet considerable evidence supports their critical functions in the nervous system, including ageing, diseases, plasticity, and regeneration (Zhao and Moore, 2018). Ageing can cause metabolic abnormalities, reduced genetic stability, and metabolic dysfunction, thereby reducing the regeneration potential of HSCs (Zhong et al., 2025). Age slows the transition of NSCs from the quiescent to the proliferative phase, leading to reduced neurogenesis and greater cognitive decline (Nicaise et al., 2020; Ruetz et al., 2024). Secretome therapy and stem cell transplantation are among the strategies that can open new frontiers in restoring NSC function and improving neurodegenerative symptoms (Zhong et al., 2025). Also, induced NSCs engineered through miR-302a have demonstrated delayed ageing and better cognitive functions in animal models, thereby offering insight into enhancing NSCs viability (Li Y. et al., 2023).

### 3.4 Induced pluripotent stem cells (iPSCs)

Pluripotent stem cells that are derived from adult somatic cells are known as induced pluripotent stem cells (iPSCs). They are reprogrammed into pluripotent cells by the stimulation of genes and factors (Ye et al., 2013). These iPSCs provide a model to study cellular mechanisms and thus contribute to understanding ageing and age-related disorders. Various studies have indicated that aged iPSCs exhibit impaired mitochondrial bioenergetics, leading to increased ROS generation. In addition to this, these stem cells have reduced mitochondrial mass and impaired mitochondrial networking as compared to the young iPSCs (Lejri et al., 2024). iPSCs can replicate various diseases pathology and help in the facilitation of the drug development process for the mimicked diseases and hence provide an upper hand in regenerative medicine approaches (Esteves et al., 2023; Jothi and Kulka, 2024).

iPSCs have also revolutionized *in vitro* research and offer great promise for regenerative medicines because of their unlimited expansion, genetic engineering, and somatic cell differentiation. The establishment of iPSCs from centenarians offers novel insights into lifespan and resistance to age-related disorders and their applications in human development and diseases, drug screening, and various cell therapies also show their growing potential in the field of regenerative medicine (Cerneckis et al., 2024; Dowrey et al., 2025).

### 3.5 Embryonic stem cells (ESCs)

Embryonic stem cells (ESCs) are unique pluripotent cells found in the inner cell mass of human blastocysts, an early stage of embryonic development. They disappear after the seventh day and form three germ layers. However, ESCs derived from blastocysts can be grown in the laboratory and proliferate indefinitely under the right conditions. These undifferentiated ESCs possess the ability to differentiate into all three germ layers (El-Hashash, 2021; National Research Council and Institute of Medicine Committee on the Biological and Biomedical Applications of Stem Cell Research, 2002). Research on human ESCs (hESCs) raises ethical concerns because the embryos used to derive ESCs are destroyed. These hESCs undergo numerous cellular, molecular, and epigenetic alterations as they age, and hence impacting their self-renewal and differentiation potential (Patni et al., 2021). In addition, hESCs can differentiate into several cell types making them possible treatment options for age-related disorders, organ failure as well as neurodegenerative diseases. Current research focuses on generating a pure hESCs-derived cell population for safer clinical applications (Whirledge et al., 2006; Yabut and Bernstein, 2011).

## 4 Stem cell ageing

Regenerative stem cells, frequently arranged in distinct niches, are found in the majority of adult organs. Stem cell function depends on interactions with the niche and is essential for tissue homeostasis and damage healing. Age-related declines in stem cells’ capacity to proliferate and differentiate into differentiated cells within the tissue are linked to a loss in the integrity and health of the tissue. Defects in maintaining stem cell quiescence, differentiation capacity and bias, clonal growth, and immune cell infiltration in the niche are among the ageing-related alterations in regenerative tissue regions. Tissue stem cell function deteriorates with age. Except for HSCs, the majority of somatic stem cells experience a decrease in both quantity and function as people age. As people age, their function declines, but their number of HSCs (as determined by phenotypic) rises. A decrease in tissue function and repair is linked to the age-dependent decline in stem cell function.

### 4.1 Intrinsic and extrinsic ageing of stem cells

Apart from the hallmarks of ageing, intrinsic and extrinsic ageing can also influence stem cells, their normal functioning, and their regenerative potential. This functional decline can occur due to internal and external agents that progress with age, leading to improper tissue maintenance and an earlier onset of age-related disorders. DNA damage, telomere shortening, and loss of protein homeostasis are among the intrinsic factors that contribute to the progressive deterioration of stem cell function. Accumulation of DDR is a major contributor to stem cell ageing, which further leads to weakening of the rejuvenation potential of stem cells (Devaraj et al., 2021; Montazersaheb et al., 2022). Similarly, the shortening of telomeres with age restricts the replicative property of stem cells and leads to stem cell exhaustion (Hambright et al., 2020). Modification in epigenetic factors alters gene expression and stem cell behaviour (Montazersaheb et al., 2022). Also, altered mitochondrial function leads to increased oxidative stress and more ROS generation, which are the major contributors to stem cell senescence and the ageing process (Tian and Dai, 2021).

On the other hand, extrinsic factors, including alterations in the stem cell niche, chronic inflammation, circulating factors like cytokines and hormones and systemic metabolic changes, also have the same effect. The stem cell niche comprises the surrounding cells and the extracellular matrix. This is necessary to maintain stem cell functioning. Age-related abnormalities in niche composition can result in weakened and impaired signaling pathways, affecting cellular behaviors and regeneration (Nalapareddy et al., 2022; Perdiguero et al., 2023). Metabolic changes and inflammation can lead to an increase in ROS and hence can initiate senescence in stem cells (Tian and Dai, 2021; Hambright et al., 2020; Montazersaheb et al., 2022). Table 1 provides a comprehensive overview of the intrinsic and extrinsic ageing of stem cells in the context of the hallmarks of ageing and their significant effects on the stem cells.

**TABLE 1:** An overview of intrinsic and extrinsic mechanisms of stem cell ageing with respect to hallmarks of ageing.

Category	Mechanisms	Major effects on stem cells	References
Intrinsic ageing	Genomic instability (DNA damage and accumulation)	Impaired self-renewal, apoptosis, and senescence	Esposto and Balendra (2022), López-Otín et al. (2013), López-Otín et al. (2023)
	Telomere attrition	Reduced proliferative ability	
	Epigenetic alterations	Identity loss, aberrant differentiation	Mi et al. (2022)
	Proteostasis loss	Accumulation of misfolded proteins, impaired functions	López-Otín et al. (2013)
	Mitochondrial dysfunction and ROS	Oxidative stress, metabolic imbalance	Picerno et al. (2021), Tian and Dai (2021)
Extrinsic ageing	Altered stem cell niche	Dysregulated signals, impaired support	Mi et al. (2022)
	Circulating factors (e.g., cytokines, hormones)	Disrupted quiescence and differentiation	
	Inflammageing	Premature activation, exhaustion	Picerno et al. (2021)
	Systemic metabolic changes	Impaired nutrient sensing, reduced regeneration	Esposto and Balendra (2022), López-Otín et al. (2013), López-Otín et al. (2023)

### 4.2 Age-related alterations in tissue homeostasis

Age-related alterations in tissue homeostasis include decreased stem cell function and increased chronic inflammation, leading to impaired tissue maintenance and regeneration. Tissue homeostasis can also be altered by different cellular and molecular changes with ageing and hence disrupts tissue equilibrium. Ageing also contributes to modification in the stem cell population as it reduces their numbers and alters the growth properties and thus impairs tissue regeneration (Ortells and Keyes, 2014). Certain stem cells, like intestinal or hematopoietic stem cells, can enhance DNA damage and decreased regenerative abilities as age progresses (Jones and Passegue, 2023). An increase in the myeloid-derived suppressor cells and regulatory T-cells leads to a condition where while protecting against severe damage, enhanced tissue degeneration also takes place (Salminen, 2021). Lymphatic efficiency can be lowered by decreased fluid balance and improper immune responses, leading to limited transfer of antigens and immune cells, resulting in weakened tissue integrity, and thus worsening the diseases associated with age (Baranwal and Rutkowski, 2019). Some studies revealed that specific changes related to age may also start tissue resilience under specific scenarios, thereby suggesting a complex relationship between tissue homeostasis and ageing (Pomatto and Davies, 2017).

### 4.3 Age-related decline of stem cells niche

**FIGURE 1:**
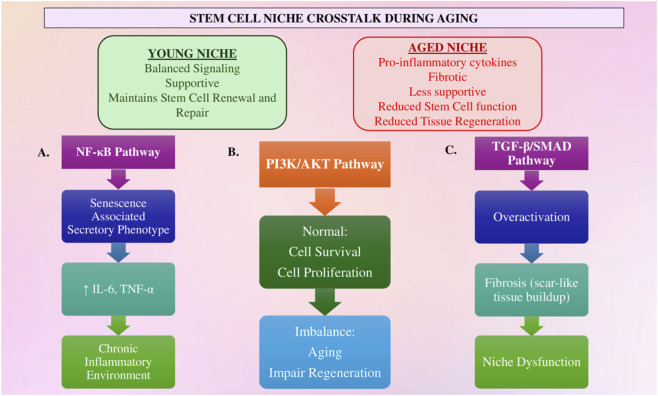
Diagrammatic representation of stem cell niche crosstalk during ageing. Stem cells reside in a niche, a supportive microenvironment that regulates their function. The diagram illustrates the contrasting features of young and aged niche, and their niche becomes disrupted by signaling pathways **(A)** NF-κB pathway which gets activated as ageing process leading to more secretion of SASP which increases pro-inflammatory cytokines thereby creating a chronic inflammatory environment; **(B)** PI3K/AKT pathways which normally enhances cell survival and proliferation whereas its imbalance during ageing leads to impairment of regenerative potential of the niche; **(C)** TGF-β/SMAD pathway which is a fundamental signaling cascade regulating various functions during embryonic development and its overactivation leads to fibrosis resulting in niche dysfunction.

Stem cell microenvironment plays as much of a role as other factors in maintaining stem cells’ health. Any loss in the stem cell niche due to ageing directly affects their functioning and tissue integrity. Due to the progression of ageing, the structural and functional abilities of the stem cell niche begin to compromise the regulatory mechanisms that are vital to the management of the stem cell growth and population. This loss is recognized by the modifications in signaling pathways, cellular interactions and spatial organization that further lead to worsening the regenerative capacity of the stem cells. The structural change can be the dislocation of niche nuclei, which results in spatial regulation of stem cells (Urman et al., 2023; Urman et al., 2024). It was observed in bone marrow that age-related declines in niche-derived factors like netrin-1, which is a critical regulator, result in the accumulation of DNA damage, further contributing to impaired functioning of stem cells by premature ageing phenotypes and impaired hematopoietic stem cell health (Ramalingam et al., 2023). The loss of niche integrity is linked with reduced stem cell activity, as demonstrated in both germline and hematopoietic stem cells, resulting in lower tissue homeostasis and regeneration capacity. Notably, the shift in germline stem cells caused by niche ageing affects their functional control, contributing to male reproductive problems (Farahzadi et al., 2023; La and Hobbs, 2020). Figure 1 shows the significant differences between the young and aged niche, along with how pathways like NF-κB, PI3K/AKT and TGF-β/SMAD can affect these stem cell niches, leading to a change in the microenvironment that ultimately results in worsening of the condition.

### 4.4 Ageing drivers specific to stem cells: an integrative perspective

While ageing hallmarks impact various cell types, their functional effects are especially evident in stem cell populations, where accumulated molecular damage directly reduces regenerative capacity. In contrast to differentiated cells, stem cells must maintain long-term genomic integrity while remaining responsive to environmental signals, making them particularly susceptible to age-related dysfunction. Recent findings suggest that stem cell ageing results from both intrinsic damage buildup and niche-mediated signalling changes, rather than from singular molecular defects. For instance, epigenetic drift and mitochondrial dysfunction hinder stem cell self-renewal, while inflammatory environments disturb quiescence and lineage equilibrium (Rossi et al., 2008; Schultz and Sinclair, 2016).

Notably, various hallmark processes have a more significant impact on stem cell populations than on differentiated tissues. Cellular senescence and inflammatory signaling in the stem cell niche diminish regenerative capacity, while systemic metabolic changes additionally affect stem cell response to repair signals (Oh et al., 2014; López-Otín et al., 2023). These findings highlight the necessity of interpreting ageing biology from a stem cell-focused perspective, prioritizing functional outcomes over mere molecular descriptions. This method improves translational relevance and facilitates focused therapeutic strategies aimed at restoring regenerative potential rather than broadly altering ageing pathways.

## 5 Stem cell-mediated anti-ageing effects: a mechanistic approach

**FIGURE 2:**
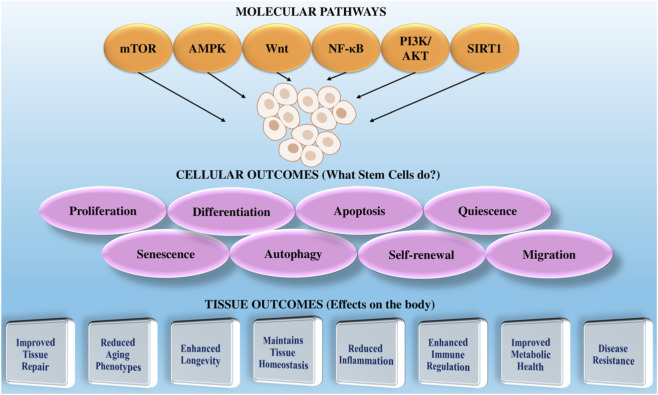
The diagram shows the integrated network of stem cell signaling pathways, their cellular responses, and tissue-level outcomes during ageing. Stem cells can be influenced by various molecular pathways, as shown in the diagram. The pathways regulate key cellular outcomes that elaborate what stem cells do at the cellular level, which together determine the stem cell health and regenerative potential. At the tissue level, these cellular responses exhibit various improved effects that enhance longevity. The figure highlights the pivotal role of stem cells in regulating and managing tissue homeostasis and resisting age-related decline.

Recent research studies demonstrated that therapeutics using various types of stem cells can slow down the progression of ageing via several complementary mechanisms (He et al., 2025). These anti-ageing effects are mainly achieved through immunomodulation, reduced oxidative stress and repair of tissue damage, contributing to various biological processes (Figure 2). As MSCs have antioxidative properties that diminish oxidative stress-related factors like superoxide dismutase and malondialdehyde in multiple organs and hence contribute to preventing age-related issues (Abumaree et al., 2017; Han and Kim, 2025; Stavely and Nurgali, 2020; Wang et al., 2024; Wang L. et al., 2023).

Stem cells release extracellular vesicles or exosomes, that carry cargo composed of microRNAs, proteins and mRNA, which restore the adjacent cells. For example, exosomes from ESCs carry high levels of miR-15b-5p and miR-290a-5p, which silence the Ccn2-AKT/mTOR ageing pathway and reverse senescence *in vitro* and in aged mouse models (Yu et al., 2023). Likewise, MSCs’ exosomes also modify recipient cells; in a senescent mouse brain model, they increase SIRT1, decrease oxidative stress and apoptosis and thus alleviate ageing phenotypes (Zhang X. et al., 2023). In skin models, MSC exosomes also attenuated oxidative stress and matrix metalloproteinase activity in fibroblast cells through the MAPK/AKT pathways, restoring fibroblast proliferation and blocking senescence (Wu et al., 2022). Therefore, the stem cell secretome communicates anti-ageing signals to the host tissue.

Stem cells can differentiate into multiple lineages to renew aged tissues. Mature MSCs, for instance, from cartilage, bone and adipose cells, replacing the senescent cells. Similarly, pluripotent ESCs or iPSCs can revive injured organs (El Assaad et al., 2024; Wu et al., 2022). Stem cells effectively reduce chronic inflammation, a significant hallmark of ageing. MSCs produce anti-inflammatory cytokines such as IL-10 and TGF-β, as well as other growth factors, which inhibit TNF-α, IL-6, and other proinflammatory mediators. This provides an anti-inflammatory region that eliminates damage and enhances repair mechanisms, contributing to homeostasis (Elmi et al., 2025; Planat-Benard et al., 2021). Furthermore, young donor adipose-derived stem cells (ASCs) have also been demonstrated to reduce immune cells and inflammation in aged mice, indicating that they secrete immune factors that modulate the immune system, thereby contributing to anti-ageing effects (Wang T. et al., 2023).

Cell-based therapies also alter tissue metabolism. Their factors enhance mitochondrial and NAD^+^/Sirtuin pathways. MSCs’ exosomes upregulate neuronal SIR1 in older mice (Zhang X. et al., 2023). Also, somatic cells reprogramming by factors like Oct4, Sox2, Klf4, and c-Myc, reverses the ageing markers; partial iPSCs reprogramming resets the epigenetic clocks, contributes to mitochondrial function and reduces SASPs and extends lifespan in progeroid mice (Li R.-L. et al., 2025). The metabolic alterations support regeneration and anti-inflammatory actions. Adipose-derived stem cells from young donors are linked to weight and abdominal fat loss in older recipients, further suggesting the role of stem cells in managing metabolic regulation (Wang T. et al., 2023). Therefore, the reduction of stem cells is very closely associated with ageing and age-related diseases. Understanding the molecular underpinnings of stem cell ageing may lead to novel therapeutic options for anti-ageing therapies (Mi et al., 2022). Recently, omics technologies have been extensively used to study age-related alterations at the molecular level, shedding light on the possibility for stem cells to reverse it (Moon and Bae, 2015).

## 6 Rejuvenation strategies

**FIGURE 3:**
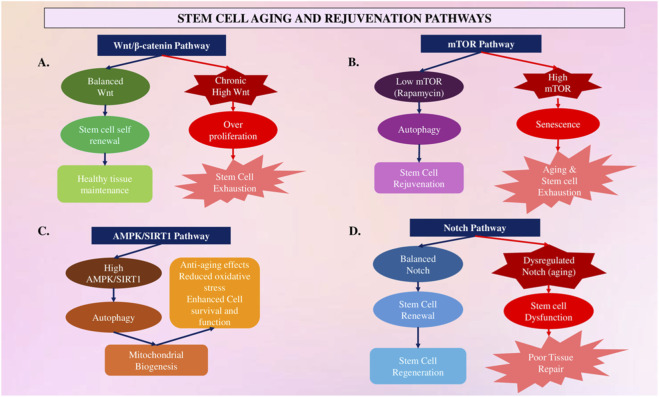
Schematic representation of major signaling pathways that regulate stem cell ageing and rejuvenation process. Under **(A)** Wnt/β-catenin pathway showing the effects of balanced Wnt signaling in maintaining stem cell self-renewal and healthy tissue maintenance, whereas chronic high Wnt signaling promotes overproliferation and stem cell exhaustion. **(B)** mTOR pathway illustrating that low mTOR activity (rapamycin-mediated) induces autophagy and stem cell rejuvenation, while high mTOR signaling contributes to senescence, ageing, and stem cell exhaustion. **(C)** AMPK/SIRT1 pathway depicting the role of elevated AMPK/SIRT1 activity in promoting autophagy, mitochondrial biogenesis, reduction of oxidative stress, and enhancement of cell survival and function. **(D)** Notch pathway showing that balanced Notch signaling supports stem cell renewal and regeneration, whereas dysregulated Notch signaling during ageing leads to stem cell dysfunction and poor tissue repair.

Rejuvenation strategies for ageing stem cells focus on restoring their regenerative capabilities, which decline with age, to improve tissue homeostasis and potentially extend health and lifespan. Various pathways regulate the rejuvenation process in the body, but impairment of these pathways can lead to poor stem cell function (Figure 3). Although the body has various pathways, recent trends have new approaches to managing stem cells via new rejuvenation strategies. The primary methods for rejuvenation strategies for stem cells include (a) preconditioning and senolytics, and (b) biomaterials and engineered niches. Environmental or chemical preconditioning can help in the regeneration of stem cells by increasing proliferation, differentiation and stress resistance (Pei, 2017; Yu et al., 2013). Hypoxic culture, growth factor priming, and expansion on youth-mimicking matrices, such as decellularised extracellular matrix, are among the techniques to boost MSCs and other stem cell types for renewal. Senolytic drugs, for example, quercetin, fisetin, and dasatinib, selectively remove the senescent cells, and thus lower SASP levels while restoring stem cell and their lineage potential. The drug quercetin showed the removal of senescent MSCs, leading to the enhancement of their proliferating and osteogenic capability, simultaneously inhibiting adipogenesis, adding other strong evidence to the senolytic approach of stem cell rejuvenation (Ji et al., 2023; Matveeva et al., 2023; Zhang et al., 2020). Additionally, MSC exosomes exhibit immunomodulatory, antioxidant and reparative potential on senescent cells, making them a source of rejuvenation as well (Wei et al., 2025).

Engineered niches like scaffolds, decellularized matrices, hydrogels, can replicate a juvenile extracellular environment to keep the stem cells viable. MSC-loaded chitosan hydrogels, along with MSC exosomes, enhanced fibroblast function, thereby increasing proliferation and collagen formation while reducing MMP/SASP factors and rejuvenating skin in older mice. Biomaterial carriers enhance transplanted cell survival by mimicking extracellular matrix signals like adhesion ligands, sequestered growth factors, and mechanical stiffness that ultimately led to better engraftment and regenerative efficiency (Elmi et al., 2025; Zhao et al., 2021). Furthermore, modifying genes is another approach that involves altering gene expressions to reverse the ageing phenotypes in the stem cells by overexpressing or knocking down specific genes. Pharmaceutical therapeutic approaches can also be used to modify the various signaling pathways involved in ageing processes. They can increase cell growth or can even decrease ageing or senescence. Various studies also demonstrated their benefits in cardiac stem cells rejuvenation applications (Kaur and Cai, 2018). In addition to this epigenetic renewal that targets cellular ageing by epigenetic clock alterations, restarting the biological ageing of cells through the expression of transient transcriptional factors. Stem cells regeneration therapies and their association with oncogenesis are a major drawback as it can worsen cancer conditions or can even cause it beforehand (Baklaushev et al., 2022).

Despite promising preclinical outcomes, there are still considerable uncertainties about the primary mechanisms responsible for MSC-mediated rejuvenation. While initial research focused on cellular engraftment and differentiation, growing evidence indicated that paracrine signaling, especially through EVs and secreted cytokines, could serve as the main therapeutic mechanism. Nonetheless, the comparative impact of these pathways continues to be debated and might differ based on tissue context, disease stage and delivery approach (Gnecchi et al., 2008; Levy et al., 2020). Elucidating these mechanisms is an essential knowledge gap that directly impacts therapeutic development and clinical application.

### 6.1 Clinical relevance of stem cell rejuvenation approaches

Stem cell rejuvenation approaches have considerable clinical significance due to their potential to address the root cause of ageing instead of merely alleviating disease symptoms. Methods like senolytic therapy, stem cell preconditioning, epigenetic reprogramming and designed biomaterial niches have demonstrated potential in revitalising stem cell function and enhancing tissue repair ability in ageing organisms. Senolytic compounds such as dasatinib and quercetin have shown targeted elimination of senescent cells, leading to enhanced tissue repair and reduced inflammation in preclinical studies. Early-stage clinical trials have indicated enhanced physical function and lower frailty-related biomarkers post senolytic treatments, emphasising their potential for translation (Justice et al., 2019; Kirkland and Tchkonia, 2020). Similarly, EVs derived from stem cells are becoming important therapeutic options as they can transport bioactive molecules without the risks associated with direct stem cell transplants. EV-derived therapies have demonstrated promising outcomes in tissue repair, neuroprotection and inflammation management, indicating their potential for clinical application in age-associated disorders (Mendt et al., 2019; Phinney and Pittenger, 2017). Stem cell delivery systems supported by biomaterials and engineered niches improve the efficiency of engraftment and cell-survival, leading to better clinical outcomes in regenerative medicine fields like skin rejuvenation, musculoskeletal repair and cardiac regeneration (Cruz-Gonzalez et al., 2025; Sun et al., 2019). Additionally, using specific transcription factors for partial cellular reprogramming has demonstrated potential in reversing epigenetic ageing indicators and rejuvenating tissue function in experimental models thereby presenting a possible approach for delaying age-related decline (Ocampo et al., 2016). In general, these rejuvenation approaches have significant potential to enhance clinical outcomes in age-related conditions such as osteoarthritis, neurodegenerative diseases, cardiovascular issues and frailty. Nonetheless, extensive randomized clinical trials and prolonged safety assessments are still crucial prior to regular clinical applications.

## 7 Tissue-specific applications

Tissue-specific stem cell applications are increasingly popular across many scientific research areas. The adult stem cells localized in certain tissues play a significant role in maintaining and repairing by replacing the damaged or senescent cells. They are stored in niches and activated by certain specific signals to multiply and differentiate when needed. However, their ability to repair certain injuries like myocardial infarction or cerebral ischemia is somewhat limited (Montagnani et al., 2016). Induced tissue-specific stem cells (iTSCs) derived from reprogrammed somatic cells have a lower tumorigenic potential, making them more suitable for regenerative medicinal purposes. These stem cells can be derived from various cell types, like hepatocytes and pancreatic cells (Saitoh et al., 2021). Moreover, stem cells can be isolated from injured tissues (post-mortem), with their regenerative potential intact. These cells have demonstrated engraft and regenerative injured tissues efficiently, giving new hope for regenerative therapeutics (Haring and Zupan, 2020). Dental tissue-derived MSCs are highly proliferative and are found to regenerate tissues of ectodermal and mesenchymal origin (Dave and Tomar, 2018). Adipose-derived stem cells are another option that is being used in the repair of tissues and, hence, managing the concerns regarding the use of ESCs (Indumathi et al., 2015). Then, resident stem cells in tissues carry out local regeneration. Further, as mentioned earlier, stem cells derive cardiomyocytes, especially those from PSCs, which revive damaged myocardium, while MSCs secrete growth factors, thereby inducing angiogenesis and recovery (Goldenberg and Carvalho, 2013; Kou et al., 2022).

In neurodegenerative diseases like PD, stem cells are used for their regenerative potential to restore functions (Liu et al., 2016). Aside from this, neural repair is also promoted by transplanted neural progenitors of iPSC origin that differentiate into neurons along with enhanced angiogenesis and neurogenesis, thereby leading to functional recovery in stroke models (Weber et al., 2025). For skin wounds, MSCs, including those derived from iPSCs, accelerate the re-epithelization process and wound closure in burn models (Farahat et al., 2025). In recent years, MSCs have also been extensively used as agents for bone and cartilage repair and have yielded promising outcomes in preclinical trials. These cells can become osteoblasts or chondrocytes, reduce bone lesions, and improve cartilage thickness in osteoarthritis patients (Hoang et al., 2022; Kou et al., 2022; Wang et al., 2020). While tissue-specific stem cells indeed promise regenerative medicine applications, proposals for therapeutics still need optimization and must be validated through stringent testing for tumorigenesis and immune rejection issues (Liu et al., 2016; Ratna et al., 2024).

### 7.1 Clinical applications

Stem cells, particularly MSCs and iPSCs, are advancing to clinical trials as regenerative therapies for tissue loss, ischemic injury, immune-mediated diseases, and other various conditions. MSCs exhibit dual benefits: they differentiate into lineages of damaged tissues and secrete immunomodulatory trophic factors and extracellular vesicles that stimulate endogenous repair mechanisms. Clinical translation approaches have demonstrated safety and early efficacy indications in cardiac repair, osteoarthritis, chronic wounds, corneal and cartilage regeneration and some ageing-related indications like frailty and skin ageing. Understanding the functional enhancements reported in clinical trials on frailty and ageing is challenging due to variability in patient groups, outcome metrics and follow-up duration. Many studies have shown that improvements in physical performance measures have been limited, leading to ongoing debate over whether the observed benefits reflect true regenerative repair or transient immunomodulatory effects (Golpanian et al., 2017; Schulman et al., 2018; Tompkins et al., 2017). Standardizing endpoints and conducting longer-term assessments are crucial for addressing these uncertainties.

However, therapeutic results vary by cell source, dose, delivery route and even manufacturing quality. Major challenges, including engraft durability, pulmonary entrapment after intravenous delivery, and trial design variability, can constitute different efficacy conclusions. Ongoing randomized, controlled trials and longer follow-ups are addressing safety, standardized manufacturing, and mechanistic biomarkers to move the applications of stem cells to the next level, meaning approved, evidence-based treatments (Garay, 2023; Margiana et al., 2022). Recent clinical trials are provided in Table 2 for better understanding of the clinical applications of stem cells in the regenerative medicinal field.

**TABLE 2:** Stem cell-based clinical trial description and status (terminated and withdrawn trials are not mentioned).

NCT number	Condition	Enrollment	Age (years)	Intervention	Status/Phase	Brief summary	Start year
NCT06877377	Ageing	60	45–65	Administration of fragmented therapeutic dsDNA	Not yet recruiting	Safety and efficacy of neural genome reconstruction technology of hematopoietic stem cells	2025
NCT06495437	Impaired Glucose Tolerance	20	35–65	Intravenous infusion of EVs from Umbilical cord MSCs (ucMSCs)	Not yet recruiting	Initial safety of human MSCs derived EVs in age related phenotype with impaired glucose tolerance	2024
NCT06501066	Pathological processes	66	65 years and older	Intravenous infusion of ucMSCs transplantation	Active, not recruiting phase I/II	Safety, efficacy, and long-term outcomes of allogeneic ucMSCs	2024
NCT06448052	Ageing, Diabetes, Inflammation, Obesity	72	40–64 years	ucMSCs transplantation	Active, not recruiting phase I/II	Safety and efficacy of transplanted ucMSCs in age-related low-grade inflammation	2023
NCT05508191	Skin ageing, Tran epidermal water loss	30	35–59 years (females)	Secretome from adipose derived MSCs (ADMSCs) via microneedle and fractional CO_2_ laser	Completed	Comparison between micro needling and laser for ADMSCs in Facial skin rejuvenation	2022
NCT05018767	Frailty	86	Child, adult, older adult	Intravenous intervention of cultured allogeneic adult ucMSCs	Recruiting phase I	Safety and efficacy for ageing frailty	2022
NCT05246813	Ageing, osteoporosis, hip fractures	24	65 years and older	Observational	Recruiting	Blood and bone marrow from hip arthroplasty patients analyzed for CHIP mutations, and single cell gene expression of hematopoietic stem cells	2022
NCT05827757	Ageing, Chronic inflammation	72	40–64 years	Autologous ADMSCs transplantation	Recruiting phase I/II	Safety after ADMSCs transplantation in inflammageing	2020
NCT04314011	Ageing Frailty	30	60–80 years	Human umbilical cord mesenchymal stem cells	Completed phase I/II	Safety test, improving health status	2020
NCT03949647	Normal Ageing	2000	18–120 years	Observational	Recruiting phase -	Change in progenitor cells with age	2019
NCT03169231	Ageing Frailty	150	70–85 years	Longeveron Mesenchymal Stem cells -intravenous	Completed phase II	Randomized, placebo controlled, double blind, parallel arm, multicentric study	2017
NCT03108898	Ageing, Esophagus Disorder	10	18 years and older	Observational	Completed phase -	Change in esophageal cells with ageing	2017
NCT03071835	Ageing Frailty, Cardiomyopathies	47	18 years and older	Observational	Completed phase -	Comparative follow up study after receiving stem cell therapies (3–13 years post treatment)	2016
NCT02065245	Frailty	65	60–95 years	Intravenous allogeneic MSCs	Completed, phase I/II	Safety of treatment with stem cells	2014
NCT01828723	Ageing, wrinkles, Lipoatrophy	6	18 years and older	Stromal vascular fraction lipoinjection	Completed phase I	Safety of stromal vascular fraction (SVF) enriched fat grafts for facial enhancement	2013
NCT01169831	Ageing, Cardiovascular Diseases	60	50–80 years	Exercise training and exercise cessation	Completed phase -	Endothelial progenitor cells regulation by short term exercise	2011
NCT00690183	Ageing	39	60–105 years	Observational	Completed phase -	Pilot study to check physical activities effect on ageing	2008

Even with the promising experimental outcomes, the application of stem cell therapies in clinical environment is still inconsistent. Although various early-stage studies show safety and feasibility, effectiveness results vary significantly across various disease contexts. For instance, advancements in musculoskeletal repair tend to be modest and affected by factors like patient age, disease progression and delivery methods (Pittenger et al., 2019). Likewise, cardiovascular applications have produced varied functional improvements, highlighting the need for standardized endpoints and prolonged outcome monitoring (Terashvili and Bosnjak, 2019). These findings emphasize the importance of differentiating between mechanistic plausibility and clinically confirmed advantages when analyzing regenerative outcomes.

### 7.2 Evidence strength and translational status of stem cell therapies

While rejuvenation strategies utilizing stem cells demonstrate considerable potential, the robustness of the evidence varies in different experimental models and clinical contexts. Many mechanistic insights come from *in vitro* systems, and small animal studies often highlight the improvements in tissue repair, inflammation regulation and functional recovery. Nevertheless, translating to human clinical results remains inconsistent due to biological complexity and differences in the study design. Clinical trials with MSCs have typically shown satisfactory safety profiles, but the therapeutic effectiveness varies significantly among different disease types. For instance, research on cardiovascular disease has indicated slight improvement in cardiac function metrics, but outcomes differ based on the method of delivery, selection of patients and protocols for cell preparation (Banerjee et al., 2018; Kahlon et al., 2018).

One major mechanistic uncertainty is whether the advantages of stem cells stem mainly from direct engraftment and differentiation or from paracrine signaling facilitated by secreted bioactive factors such as EVs and cytokines, Growing evidence supports the paracrine hypothesis, therefore, indicating that the modulation of inflammation and tissue repair through secretome may be primary therapeutic mechanism (Gnecchi et al., 2008; Levy et al., 2020). Another important key factor is the variability of clinical results among trials. Differences in stem cell origin, growth methods, dosage approaches and outcomes measures greatly affect the reported effectiveness. Numerous studies are still in Phase I or Phase II, concentrating mainly on safety instead of a clear therapeutic advantage (Trounson and McDonald, 2015). Consequently, although stem cell rejuvenation approaches demonstrate significant translational promise, existing evidence supports measured optimism, highlighting the necessity for standardized methods, extensive randomized trials, and prolonged follow-up to validate therapeutic efficacy as well as safety.

## 8 Manufacturing, ethical and regulatory investigations

Emerging advances make it necessary to navigate the manufacturing, ethical and regulatory investigations to provide safe and effective therapeutics. Quality consistency in manufacturing stem cell-based products, including good manufacturing processes (GMP) and chemistry, manufacturing, and controls (CMC) regulatory guidelines to maintain reliability of product efficacy, is of foremost importance (Andrews et al., 2014; Beetler et al., 2023). The variability among differentiated cell types poses a significant challenge in accomplishing smooth and uniform therapeutic results (Jha et al., 2021). Ethical concerns include the source of donor tissues, the methods and technologies involved in the reprogramming, the use of pre-existing stem cell lines with proper consent and ownership of the obtained biological materials. This further extends to a broader concept which consists of legally informed consent documents, fair and equal access, as well as unverified stem cell trials, as all these together can cause problems and patient safety concerns. No compromise in these aspects is tolerated (Andrews et al., 2014; Jha et al., 2021; Khabade et al., 2024).

Different countries have their own regulatory bodies to regulate and manage stem cell therapies so that no inconsistencies will be there in safety and effective standards (Singh and Pant, 2022). Regulatory bodies like the Food and Drug Administration (FDA) and the European Medicines Agency (EMA) have established fast-track processes like Regenerative Medicine Advanced Therapy (RMAT) to balance innovation with safety issues (Jayaram et al., 2025). Cases like Holocar ®, which is an EU-approved limbal stem graft, underwent thorough examination, whereas an unlicensed US clinical body operated without any regulation (Eder and Wild, 2019; Jayaram et al., 2025). According to National Guidelines for Stem Cell Research, India’s ICMR/CDSCO regulations also require GMP/GTP-compliant manufacturing, NABL certifies GLP/GMP laboratories and multiple-tier ethics review (ICSCR, IEC, CDSCO) with rigorous QC (sterility and potency) and safety testing, like risk of teratoma for cell products. The ongoing evolution of global and national standards involving regulators, ethicists and patient advocates is promoted to achieve the safe, responsible and evidence-based implementation of regenerative therapeutics. Table 3 shows the major regulatory bodies and their prime motives to smooth the regulation of stem cell management.

**TABLE 3:** A summary of existing regulatory bodies (both Indian and international) with their objectives for the management of stem cell research.

S. no.	Regulatory body	Primary agenda/Objectives	References
1	International Council for Harmonization (ICH)	• Harmonize pharmaceuticals technical requirements (GCP/CMC) region-wise to allow global development and quality benchmarks for biologics/ATMPs	Iglesias-López et al. (2019)
2	Internation Society for Stem Cell Research (ISSCR)	• Publish field specific ethical and clinical translation guidelines for human stem cell research and clinical trials	Lovell-Badge et al. (2021)
3	US Food and Drug Administration (FDA)	• Regulate cell and gene therapies through IND/BLA pathways • Provide expedited/regenerative pathways (RMAT) and comprehensive CMC guidelines • Long term follow-up	U.S. Food and Drug Administration (2025)
4	European Medicines Agency (EMA/CAT)	• Assess ATMPs via CAT/CHMP • Guarantee quality/safety/efficacy of advanced therapies • Facilitate centralized marketing authorization	Kassim and Somerville (2013)
6	Medicine and Healthcare products Regulatory Agency (MHRA, UK)	• Evaluate ATMPs and organize clinical trial authorization • Post market safety in the UK	Brigden (2024)
7	Pharmaceuticals and Medical Devices Agency (PMDA, Japan)	• Control regenerative medicine under PMD/ASRM acts • Facilitate conditional/time-limited approvals and separate RM pathway with focus on early access with post marketing data	Tobita et al. (2016)
8	National Medical Products Administration (NMPSA, China)	• Classify and regulate cell and gene therapies and tissue engineered products • Offer pathways and product classification According to national drug and device paradigms	Yoon et al. (2024)
9	Central Drugs Standard Control Organization (CDSCO) and Indian Council of Medical Research (ICMR)/Department of Biotechnology (DBT), India	Integrated framework: • CDSCO takes care of regulatory approvals • ICMR/DBT offer National Guidelines for Stem Cell Research	Jotwani and Sinha (2017)
10	Therapeutic Goods Administration (TGA, Australia)	• Regulate biologicals and ATMPs • Institute GCP.GMP standards • Establish avenues for conditional approvals and clinical trial monitoring	Yoon et al. (2024)
11	Health Canada	• Regulate advanced therapeutic products (ATPs) • Ste policy stances on autologous cell therapies • Update regulatory tools to ensure balance between innovation and safety	Government of Canada (2020)
12	National Health Surveillance Agency (ANVISA, Brazil)	• Classify and authorize ATMPs, sanitary registration, GMP and clinical regulations to promote safe clinical translation gene or cell therapies	Silva Junior et al. (2021)
13	Ministry of Food and Drug Safety (MFDS, South Korea)	• Take national regulatory Leadership on cell- or gene-based therapies and tissue engineered products • Make requirements consistent with regional best practice	Yoon et al. (2024)
14	Health Sciences Authority (HSA, Singapore)	• Assess cell and gene therapy clinical trials and market authorization • Stress risk-based regulation and good manufacturing practice	Yoon et al. (2024)

## 9 Challenges

**FIGURE 4:**
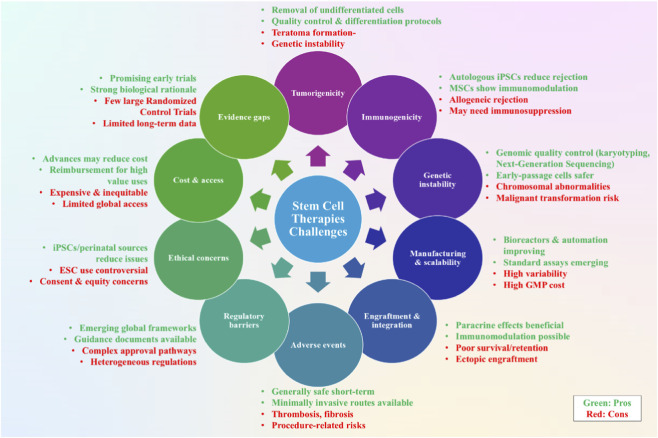
The diagram summarizes the major challenges of stem cell therapies along with their advantages and limitations. The radial diagram highlights the major challenges faced in stem cell therapies and beside each challenge, their pros (in green) and cons (in red) are also shown to emphasize both the promise and recent barriers in translating the stem cell research into safe, and effective clinical interventions for the betterment of mankind.

Stem Cell therapies have increasingly been considered as a means to reverse ageing. However, these therapies offer numerous challenges for their translation into the clinical environment (Figure 4). There are certainly some safety concerns, for example, PSCs can form teratomas, tumors or abnormal tissues if the differentiation somehow remains incomplete or occurs incorrectly (Hoang et al., 2022). Even after safety assurance, variability in efficiency is still a problem that needs to be managed. Most clinical trials showed minimal to moderate improvement, if any, and thus raise questions about whether the improvement comes from paracrine signaling or the true tissue regeneration process. Ageing also disrupts stem cells and their functions by inducing senescence, loss of growth potential and accumulation of DNA damage. This further lowers the regenerative capacity of both autologous and allogenic cells (Garay, 2023). As age plays a significant part in regulating stem cells’ efficiency, the age of stem cell donors is very crucial. Younger donors are mostly acceptable, but this brings up immunological concerns (Atkinson, 2019; De et al., 2020). Turning this strategy into a therapy raises more problems related to manufacturing and standardization. Producing iPSCs or ESCs-based products under GMP-compliant conditions needs adherence to strict quality standards, necessary infrastructure, and high-priced technologies (Francis et al., 2025). On the safety side in the longer run, immunogenicity, immune rejection, off-target engraftment, and senescent cells introduced through therapy potentially remaining while inducing prolonged inflammation and consequently neutralizing positive outcomes, are to be considered (Luo et al., 2025).

Aside from biology, there are ethical, regulatory, and societal concerns as well that need to be overcome. As quoted in the previous section, the sourcing of stem cells is a moral controversy, whereas incompatibility in international regulatory structures induced the development of untested stem cell clinical trials that offer risk and untested treatment. Still further, concerns around equitable access and affordability will limit such availability, threatening to become a primary cause of health disparity among aged populations. Therefore, in principle, stem cell therapies hold great promise for regenerative medicine in ageing, but their deployment requires improvements in safety, efficacy, scalability, manufacturing, and ethical regulation.

## 10 Limitations

The limitations of existing stem cell research and its clinical trials are multifaceted, encompassing ethical, logistical, and scientific challenges (Figure 4). Despite the potential of stem cell therapies, there exist significant barriers that limit practical clinical application. These limitations can be divided into some major areas: (a) trial design and execution issues, (b) scientific and technical challenges, and (c) ethical and legal concerns. A substantial number of clinical trials are poorly designed, with tiny participant sizes and a lack of randomization and blindness, compromising the reliability of results. Most of the trials are in early phases, which indicates a lack of progression to more advanced research that could validate efficacy (Ji et al., 2022). Moreover, many trials have demonstrated modest efficacy, especially in complex diseases like stroke, where the pathophysiological mechanism of the disease restricts the treatment success. The short half-life of various bioactive proteins secreted by stem cells and their short differential potential further limit the therapeutic outcomes (Bang et al., 2016; Pastorello and Slevin, 2023).

As already mentioned in Section 9, ethical considerations arise mainly from the source of stem cells, especially ESCs, which lead to legal issues, restrictions in various jurisdictions and raise ethical dilemmas due to the destruction of embryos, resulting in limitations in their clinical applications. Also, the moral indications of research on stem cells frequently result in inconsistent regulations among various countries and thus complicate international collaborations, which are necessary for better ethical management (Volarevic et al., 2018; Yadav et al., 2024). The informed consent process also gets complicated as patients also face therapeutic misconceptions by believing that experimental treatments are guaranteed to be effective. Furthermore, people are also concerned about equal access to all the available stem cell therapies, particularly those belonging to marginalized populations, which ultimately raises more ethical questions regarding fair access to healthcare (Jayaram et al., 2025).

Safety concerns are equally important in terms of limiting the utilization of stem cells in regenerative medicine. The potential of stem cells to promote tumor growth (tumorigenicity) and metastasis is a major safety factor in all stem cells, especially with MSCs, as they are rigorously used in stem cell research (Volarevic et al., 2018; Yadav et al., 2024). Following GMP standards is crucial for ensuring safety and monitoring of stem cell products, yet compliance remains a challenge for researchers. Lastly, the long-term safety of stem cell-based therapies is still under study, which requires vigorous pre-clinical analysis (Li F. et al., 2023).

While these limitations have significant value, researchers are still advancing ongoing studies on stem cell technology and combinatorial therapeutic approaches. Altogether, they may eventually address these limitations, leading to more effective treatments in the future. Also, regulating new approaches with patient safety and ethical issues remains a crucial part of advancing stem cell research and its applications.

### 10.1 Strengths and limitations of the review

This review has various significant advantages that boost its scientific importance. It first combines recent developments in cell research with new rejuvenation approaches offering a thorough insight into the association between stem cell impairment and ageing processes. Additionally, the inclusion of clinical trial data reinforces the translation viewpoint and emphasizes current initiatives aimed at the therapeutic use. Moreover, the analysis systematically links molecular characteristics of ageing with regenerative strategies and thus providing mechanistic understanding for therapeutic targeting.

In spite of these advantages, there are some limitations that should be acknowledged. The review mainly relies on published studies which could lead to publication bias and limit the addition of unpublished or negative results. Also, the rapidly changing landscape of regenerative medicine indicates that certain new therapies may still lack adequate clinical validation. Additionally, differences in stem cell sources, delivery techniques and trial methodologies across research hinder the capacity to reach uniform conclusion about safety and effectiveness (Mahla, 2016; Trounson and McDonald, 2015). Another limitation lies in the heterogeneity of ageing processes among individuals and tissues, making it difficult to translate experimental results into standard clinical treatments. Future systematic reviews and meta-analyses that include standardized outcome measures are needed to strengthen evidence based clinical decisions in stem cell rejuvenation therapies.

## 11 Future perspectives

Regenerative medicines are advancing day by day and hence offer a ray of hope for utilising stem cell therapies to tackle the biological processes that contribute to ageing. These therapeutics take advantage of stem cells’ unique potential for self-renewal and differentiation into different cell types to help reduce age-related decline. Short and easily accomplishable future goals include strengthening one’s own cells through partial reprogramming, a part of bioengineering, and the use of drugs that have the capability to modulate senescence. This approach aims to recover cell potency while combining therapeutics that involve the senolytic technique and immunomodulatory ability to improve the graft procedures and post-operation conditions, thereby reducing the pain (Kang, 2019; Kaur et al., 2022; Lelarge et al., 2024; Wyles et al., 2024). Improving *in vivo* reprogramming and targeted delivery with an engineered niche could help with organ-level regeneration even without the need for widespread cell transplantation (Kirkeby et al., 2025). Recent systems-level models utilizing cell-state landscape dynamics and framework of epigenetic fidelity indicate that ageing and rejuvenation might represent transitions among stable cellular states, offering novel conceptual directions for stem cell-oriented regenerative approaches (Yücel and Gladyshev, 2026; Memczak et al., 2025). Scalable GMP-graded manufacturing of iPSCs, automated analysis, and reliable potency assays are vital for turning the preclinical studies of good results into clinical trials or final products (Francis et al., 2025). Current clinical trials are mainly about the safety and efficacy of stem cell therapies in age-related patient populations, with better findings (El Assaad et al., 2024). As we learn more about stem cells and their biology, these therapeutics may become more widely used anti-ageing treatments, ultimately leading to breakthroughs in extending life and improving the overall quality (Chang et al., 2022). However, fair and evidence-based uses will need ethical oversight and standard regulations, along with strong multi-site trials.

Additionally, genetic and epigenetic engineering, like CRISPR/Cas9, allows for specified alterations in stem cells in order to promote longevity, like developing resistance to ageing or supporting the expression of therapeutic factors. However, CRISPR engineering can trigger ageing and inflammation in hematopoietic stem cells, which raises safety-based questions (Conti et al., 2025). Stem cells can also be transformed into organoids or bio-printer 3D tissues that include vascular structures (Huang et al., 2025; Lancaster and Knoblich, 2014). Integrated single-cell omics with artificial intelligence (AI) can help unravel cellular variability, predict ageing patterns, and simultaneously develop personalized ageing clocks (Xie et al., 2025; Song et al., 2018). Stem cells can also be used with other treatment regimens, for instance, MSCs with nanocarriers, or administering senolytics before the infusion of stem cells to clean specific regions. This approach positions stem cell therapies as a key strategy for better promotion of longevity (Barzilai et al., 2018).

Future research should focus on empirically testable models that specifically address unresolved disputes. For instance, comparative analyses measuring engraftment efficiency against secretome activity in standardized disease models could elucidate key therapeutic pathways. Likewise longitudinal clinical trials that include molecular biomarkers of biological ageing, like epigenetic clocks or inflammatory profiles, could offer objective assessments of treatment effectiveness. These predictive methods might assist in differentiating transient symptomatic relief from sustained regenerative outcomes (Horvath and Raj, 2018; Justice et al., 2019).

## 12 Conclusion

The phenomenon of ageing weakens the natural regenerative and repair mechanisms of the body, leading to a decrease in the quality of life and longevity. This has given rise to regenerative medicinal approaches that focus on rejuvenation techniques rather than simply taking care of repair mechanisms, thus relying on self-renewal and remarkable differentiation potential of stem cells. Ageing can even decline the functionality of the native stem cells, and hence, engineered, and healthy stem cells come to the rescue as they have the potential to regain the functions. Additionally, earlier experiments in various diseased conditions like PD and frailty, which are most common in the aged population, demonstrated that the stem cell therapeutic approach can improve the quality as well as resilience of life. Still, there are various challenges in fully unlocking the potential of stem cells in regenerative medicinal approaches. Long-term outputs for better human health still need to be studied. According to clinical trials as well, most of the studies are either in phase I or phase II, indicating an urgent need for better research on this new therapeutic.

The next step for better investigations is to improve the understanding of stem cell biology and its role in modulating ageing at the gene level, improve cell engineering to avoid ageing and increase therapeutic specificity. To quantify biological age and monitor treatment efficacy, it is necessary to develop valid and reliable biomarkers. Another necessary requirement for the safety and efficacy testing of newly developed products from stem cells is well-designed clinical trials on a large scale. The final goal of stem cell therapies is to investigate the mechanisms behind ageing itself and treat them from the core rather than treating diseases one at a time. Gradual improvements like slowing down organ deterioration can contribute to increased lifespan and improved mobility in older adults. With the right care and well-implemented measures, these approaches can become an essential part of medicine for elderly people and hence help them to stay stronger, healthier, and more independent.
